# Patterns of Ethambutol Ocular Toxicity in Extended Use Therapy

**DOI:** 10.7759/cureus.4408

**Published:** 2019-04-08

**Authors:** Salil Mehta

**Affiliations:** 1 Ophthalmology, Lilavati Hospital, Mumbai, IND

**Keywords:** ethambutol, toxicity, perimetry, optic neuropathy

## Abstract

We report the clinical and perimetric findings of three patients with ethambutol toxicity with an extended use regimen. In this observational case series, we extracted data from case records that included age, sex, complaints, details of the current disease, anti-tubercular therapy, ocular findings, and perimetric findings. We identified three patients, two female and one male with ages ranging from 16 to 65 years (mean: 37 years). The perimetric patterns we observed were incomplete homonymous hemianopia in two patients and bitemporal hemianopia in one. Incomplete hemianopia was the predominant perimetric finding, suggesting that the likely lesions were chiasmal or post-chiasmal rather than pre-chiasmal.

## Introduction

Ethambutol-induced visual complications (reduced visual acuity, dyschromatopsia, and scotomas on perimetry) though well reported, are rare. Current guidelines now mandate greater durations and doses, and this increases the risk of ocular complications. We report the clinical and perimetric findings of three patients on extended use ethambutol therapy.

## Case presentation

Case one

A 16-year-old female patient presented to us with bilateral reduction of vision since the last 10-12 days. She first presented to the emergency department with complaints of severe headache and fever since the preceding two-three days. She underwent a detailed systemic evaluation. Magnetic resonance imaging (MRI) scan at this time showed multiple ring-enhancing lesions in the brain and a chest computed tomography (CT) showed tiny miliary nodules scattered throughout both lung fields. She was diagnosed as a case of miliary tuberculosis (pulmonary and cerebral). Dilated fundus exam revealed yellow-white choroidal lesions consistent with miliary tubercles. She was started on first-line anti-tubercular treatment with rifampicin (450 mg/day), ethambutol (800 mg/day), pyrazinamide (1500 mg/day), and isonicotinylhydrazide (INH; 300 mg/day).

Two months later, following a poor clinical response and a drug sensitivity test (DST), she was started on kanamycin, moxifloxacin, ethionamide, and clofazimine in addition to the earlier drugs. 

A worsening headache led to a repeat MRI scan a month later that detected a leptomeningeal enhancement. Subsequently, linezolid was started but was withdrawn a month later due to gastric intolerance. Following five months of treatment of ethambutol, she noticed the onset of reduced vision and was referred to us. 

On examination, her best-corrected visual acuity was 6/60 in the right eye and 6/120 in the left. Extraocular movements were normal as was her anterior segment examination, including her pupillary reactions. Dilated fundus examination revealed normal findings in the right eye and normal disc and macula with a single choroidal tubercle along the superotemporal arcade in the left eye. 

**Figure 1:**
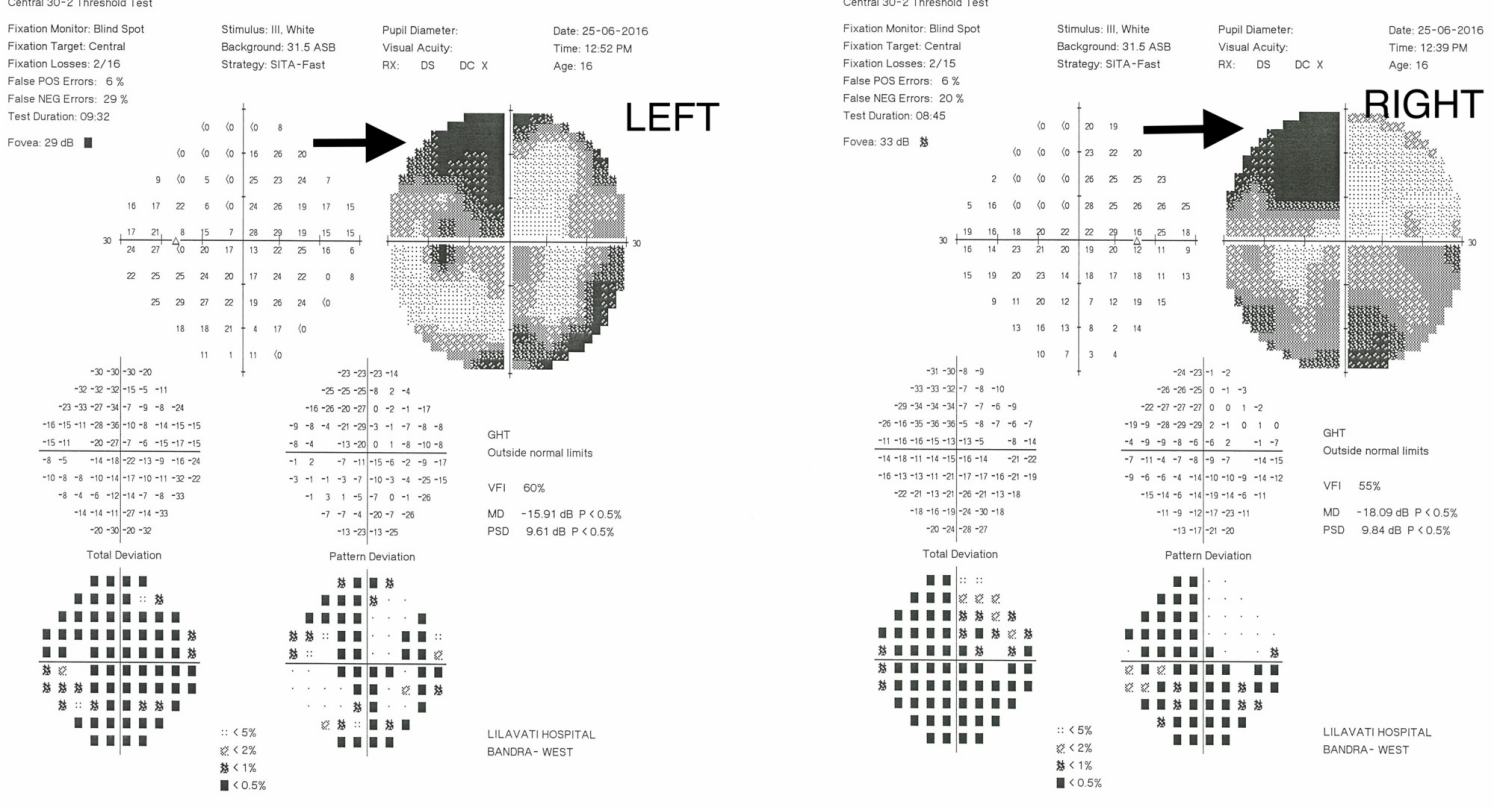
The perimetric features of case one showing incomplete left homonymous hemianopia (arrows)

She underwent a visual field examination (central 30-2, SITA-Fast), which revealed an incomplete left homonymous hemianopia with additional defects in the inferior quadrants of the right eye (Figure 1). 

The specific clinical picture suggested ethambutol toxicity, which was then stopped and she was advised to follow-up. At last follow-up (three months), her visual acuity had returned to normal and she was referred back to her physician. 

Case two

A 65-year-old male patient presented with complaints of bilaterally reduced vision. He had been investigated and been treated earlier for small cell cancer of the lung and had undergone chemotherapy for the same. Seven months earlier, following persistent breathlessness, he underwent radiological investigations and was found to have a pleural effusion. A routine examination of the pleural fluid revealed mycobacterial infection of the pleura that was the treated with rifampicin (450 mg/day), ethambutol (800 mg/day), pyrazinamide (1500 mg/day) and INH (300 mg/day). Following drug-induced hepatitis, the regimen was reduced to rifampicin and ethambutol with the addition of levofloxacin. 

**Figure 2:**
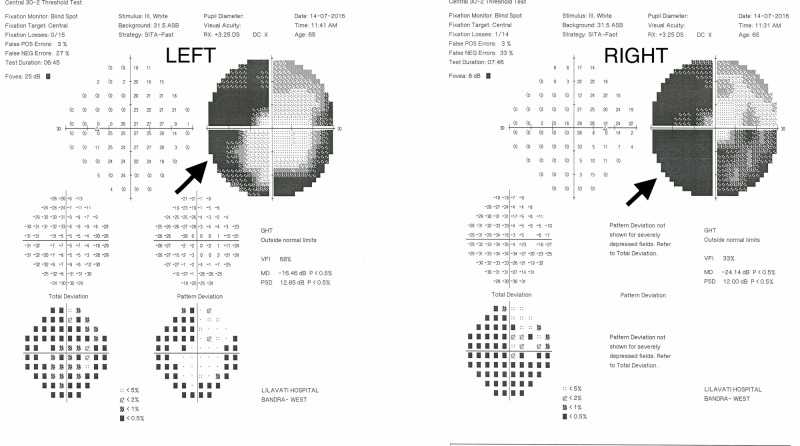
The perimetric features of case two showing incomplete left homonymous hemianopia (arrows)

On examination, his best-corrected visual acuity was 6/15 in either eye. The extraocular movements and his anterior segment examination were normal. Dilated fundus examination was normal. He underwent routine testing. A visual-evoked potential (VEP) testing showed reduced amplitude and prolonged latency on either eye, suggestive of bilateral optic axonopathy. An MRI scan revealed multiple round peripherally enhancing lesions in both cerebral hemispheres and in the right cerebellum. Central 30-2 perimetry revealed an incomplete left homonymous hemianopia with additional inferior temporal defects in the right eye (Figure 2). A diagnosis of ethambutol toxicity was made, as the lesions in the cerebral hemispheres were not consistent with the perimetric findings.

The patient declined all further treatment and was lost to follow-up.

Case three

A 30-year-old female patient presented to us with complaints of blurred vision for a month. Eight months earlier, she had undergone a routine uncomplicated laparoscopic myomectomy. Two months following this procedure, she revisited the gynecologist with complaints of abdominal pain and a feeling of hardness over the anterior abdominal wall. An ultrasound examination supplemented with an MRI study revealed fluid collections along the subcutaneous and muscular plane with adjacent subcutaneous edema. A fine needle aspiration cytology (FNAC) obtained tissue sample revealed a necrotizing granulomatous inflammation of likely mycobacterial origin. She was started on an antituberculous regimen consisting of rifampicin (450 mg/day), ethambutol (800 mg/day), and additional clarithromycin (1000 mg/day) to cover possible atypical mycobacteria. 

Five months after starting this regimen, she started noticing “blurring and haziness” in both eyes. She consulted an ophthalmologist who noted normal visual acuities bilaterally (6/6), normal appearing anterior segments and fundi. Her color vision, as tested on Ishihara plates, was found to be normal as well. As a precautionary measure, her ethambutol was discontinued. Her symptoms persisted and she sought a second opinion. 

**Figure 3:**
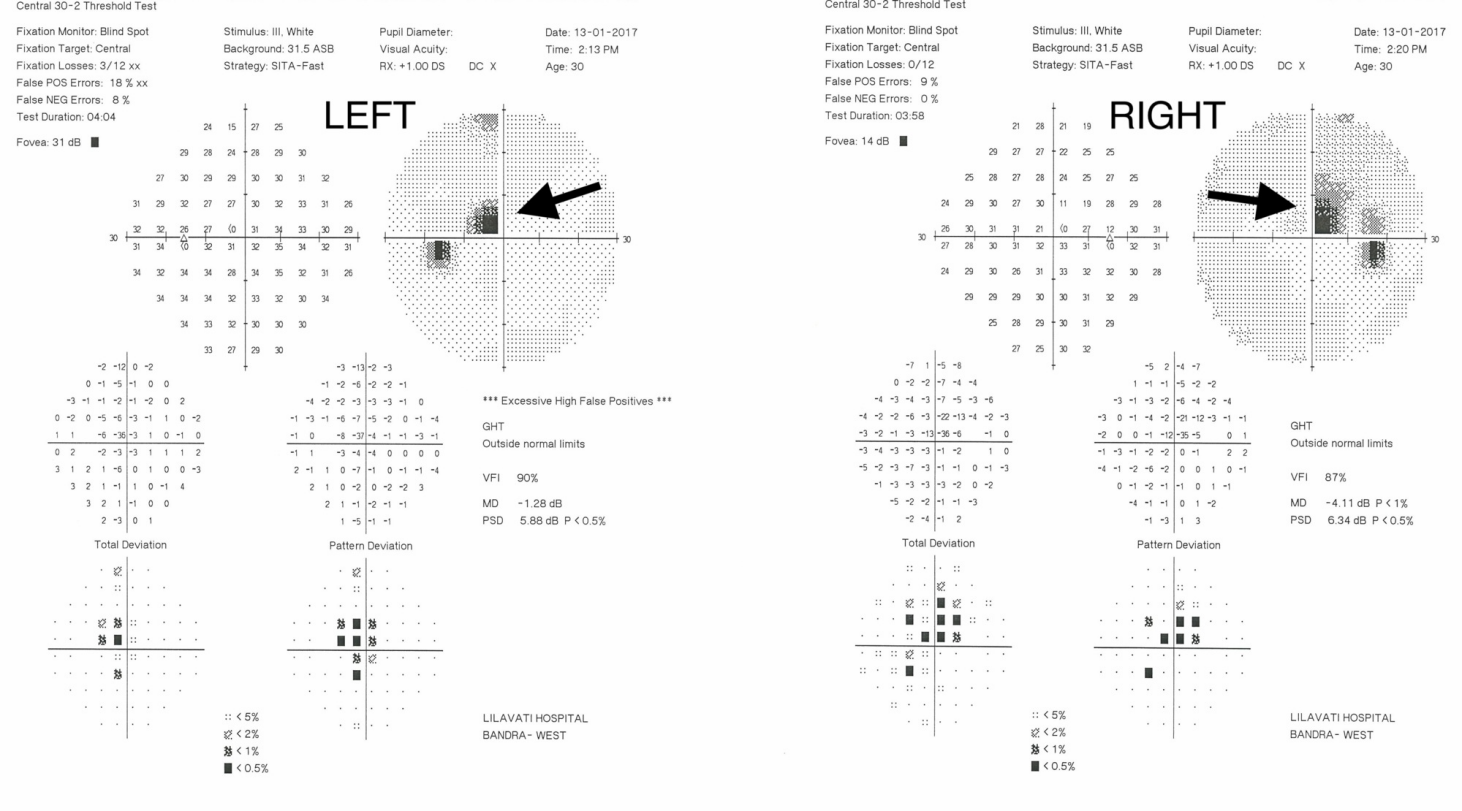
The perimetric features of case three showing bitemporal hemianopia (indicated by the arrows)

On examination, her visual acuity was 6/12(R) and 6/12 (L). Her anterior segments and fundi were normal appearing as were her pupillary reactions. She underwent baseline perimetry (central 30-2, SITA-fast), which revealed central bitemporal hemianopia that respected the vertical midline, consistent with ethambutol toxicity (Figure 3). 

Ethambutol treatment was discontinued and she was advised to undergo a regular follow up with her primary physician. At last follow-up, a month later, she was visually asymptomatic and her visual acuities were 6/6 bilaterally. She declined further field testing.

## Discussion

Ethambutol is a bacteriostatic drug that is routinely used for the treatment of all forms of typical and atypical mycobacterial infections. It works by disrupting arabinogalactan synthesis through the inhibition of arabinosyl transferase. This obstructs the formation of the cell wall mycolyl-arabinogalactan-peptidoglycan complex and leads to increased permeability of the cell wall [1]. Its toxic effects may be related to this chelating action and its subsequent RNA synthesis inhibition. This inhibition may extend to mammalian cells and especially the mitochondrial DNA. Apart from anecdotal idiosyncratic reactions, this toxicity is largely dose and duration dependent [2].

The recommended dose of ethambutol has traditionally been 15 mg/kg daily for the first two months of any anti-tuberculosis regimen, as was suggested initially by the WHO (World Health Organization). Its ocular complications were noted early on and are described as a reduction in visual acuity, dyschromatopsia, and scotomas on perimetry. These did and still do constitute the major adverse event during ethambutol use. In a meta-analysis, Ezer et al. noted, from the available studies, that patients enrolled in two-month duration regimens with drugs dosages from 15-20 mg/kg/day appeared to have a low rate of visual complications. Of the two studies analyzed, permanent impairment was noted in none of 52 (0%) and five of 72 (7%) patients. Overall, the rates of any form of visual impairment or permanent visual morbidity were 19.2 and 2.3/1000, respectively, in patients on dosages up to 27.5 mg/kg per day for 2-9 months [3]. Significant visual improvement was noted in the majority of patients on cessation of treatment. 

More recently, the Revised National Tuberculosis Control Program (RNTCP) in India has suggested an extension of duration for ethambutol for up to six months for new cases (2HRZE + 4HRE) and for 24-27 months in cases of MDR (multi-drug resistant tuberculosis) [4]. Additionally, there is an increasing trend for physicians to use higher doses of ethambutol (up to 1200 mg/day), especially in drug-resistant tuberculosis. An increase in both these variables is likely to significantly increase the risk of ocular complications. 

Visual morbidity commonly manifests as complaints of blurred or reduced vision, reduced color perception or any combination of this. Evaluation of these patients usually reveals normal anterior and posterior segments, reduction in visual acuity, dyschromatopsia (on Ishihara plates or Farnsworth Munsell 100 Hue test) and scotomas on perimetry. 

Perimetry provides a safe and accurate way to assess the effect of ethambutol on the visual system. Specific perimetric patterns suggest intensity and the site of the lesion. Ethambutol-induced neuropathy commonly results in bilateral and symmetric central/cecocentral scotomas, possibly due to ganglion cell toxicity. A significant proportion of patients may demonstrate bitemporal hemianopic central scotomas as a result of posterior chiasmal compression. These defects usually respect the vertical midline and may be caused by lesions in the crossed chiasmal fibers [5]. Various authors have suggested that the primary lesion is an optic nerve involvement (as suggested by a central/cecocentral scotoma) or a chiasmal or a post chiasmal lesion (suggested by hemianopia)[6]. 

We describe the clinical and perimetric findings of three patients. These include two women and one man with ages ranging from 16 to 65 years. The dosages were 800 mg/day but had durations of 5.5 and 7 months, as suggested by the newer guidelines. In these six eyes, the visual acuities ranged from 6/12 to 6/120 and in all these patients, the examination of the anterior and posterior segments was normal. There was no color vision abnormality seen in one patient (case 3) but the other two were not tested. VEP abnormalities suggestive of bilateral optic axonopathy were seen in one patient (case 2), but we were unable to conduct this test in the other two due to resource restrictions.

The perimetric patterns we observed (cases 1 and 2) were incomplete homonymous hemianopia in two patients associated with inferior quadrantic defects. This tends to suggest a post-chiasmal lesion. The third pattern (case 3) is bitemporal hemianopia, suggesting a chiasmal lesion. We found no central scotomas in our patients. 

A bitemporal pattern has rarely been reported. Osaguona et al reported one such case and used magnetic resonance imaging (MRI) to demonstrate hyperintensity within the chiasm on axial T2 imaging [6]. Kho et al. reviewed 19 cases of optic neuropathy due to ethambutol and found that the majority (36 of 38 eyes) had visual field loss predominantly in the temporal field. Of these, 31 had identifiable margination along the vertical midline (often with central scotomas) and 12 eyes had bitemporal defects, suggesting that these defects are commoner than suspected [7].

## Conclusions

This observational case series reports the clinical and perimetric findings of three patients with toxicity who were on treatment with an extended ethambutol regimen. Their visual acuities ranged from 6/12 to 6/120 and all these patients had normal baseline ophthalmic examinations. On perimetric analysis, the patterns we observed were incomplete homonymous hemianopia in two patients associated with inferior quadrantic defects and bitemporal hemianopia in the third. With the now recommended increases in the dosage and duration of ethambutol therapy, ocular toxicity is likely to be more common and physician need to be aware of these less commonly described but nonetheless distinct perimetric patterns. 
